# Supplementary material for: Disentangling the effect of host-genotype and environment on the microbiome of the coral Acropora tenuis
Source: PeerJ. 2019 Feb 5;7:e6377. doi: 10.7717/peerj.6377 (PMC6368029; doi:10.7717/peerj.6377)
Supplement: Supplemental Information 1 [file peerj-07-6377-s001.docx]

**Disentangling the effect of host-genotype and environment on the microbiome of the coral *Acropora tenuis***

**Equation S1.** Equation for a) Maximum quantum yield and b) Chlorophyll *a* concentration:

a) Maximum quantum yield (*F_v_/F_m_*) was calculated by measuring the minimum (*F_o_*) and maximum (*F_m_*) fluorescence of the Symbiodiniaceae within the coral host tissue.

$$F_{v}{/F}_{m}=(F_{m}-F_{o})/F_{m}$$

b) Chlorophyll *a* concentrations were calculated using the following equation:

$$Chlorophyll a (\mu g ml^{-1})=11.47 \times A_{663}-0.64 \times A_{630}$$

**Table S1.** 16S rRNA gene amplification using primers 27F and 519R.

**A)** Preparation of master mix for amplification of 27F and 519R region of the 16S rRNA gene

| **Component** | **Volume (µl)** | **Final concentration** |
| --- | --- | --- |
| 10 x Immolase Buffer | 2.5 | 1x |
| 10 mM dNTP | 0.5 | 200 nM |
| 50 mM MgCl_2_ | 1.25 | 2.5 mM |
| ILM_27F Uv3 (forward) (5µM) | 2.5 | 500 nM |
| ILM_519R (reverse) (5µM) | 2.5 | 500 nM |
| Immolase DNA Polymerase (5U/µl) | 0.2 | 1 Unite |
| H_2_O | 14.55 | - |
| Template | 1 | - |
| Total Volume | 25 | - |

**B)** Thermocycler conditions for the amplification of 27F and 519R region of the 16S rRNA gene)

|  | **Temperature (°C)** | **Time (mm:ss)** |
| --- | --- | --- |
| Activation | 95 | 10:00 |
| Amplification (35 cycles) | 94 | 00:30 |
|  | 55 | 00:10 |
|  | 72 | 00:45 |
| Final Extension | 72 | 10:00 |

**Table S2.** Statistical output of the TukeyHSD post hoc test (95% confidence interval) used to compare a) protein concentration, b) photochemical efficiency and c) Symbiodiniaceae density between treatment groups (control, acute stress and cumulative stress).

**a) Protein concentration**

diff lwr upr p adj

cumulative stress-control -0.62728959 -1.1800774 -0.07450175 0.0220395

single stress-control 0.08027902 -0.4885350 0.64909302 0.9397306

single stress-cumulative stress 0.70756861 0.1547808 1.26035646 0.0083073

**b) Photochemical efficiency**

diff lwr upr p adj

cumulative stress-control 1.0403706 0.5161774 1.5645637 0.0000231

single stress-control 0.3404764 -0.1989139 0.8798667 0.2942703

single stress-cumulative stress -0.6998942 -1.2240874 -0.1757010 0.0055985

**c) Symbiodiniaceae** **density**

diff lwr upr p adj

cumulative stress-control -0.6436856 -1.1836395 -0.1037318 0.0151617

single stress-control 0.2429778 -0.3126302 0.7985857 0.5530122

single stress-cumulative stress 0.8866634 0.3467095 1.4266173 0.0005010

**Table S3.** Statistical output of PERMANOVA (adonis2, vegan package) testing the effect of treatment, sampling time point and tank on the microbiome composition within each host genotype (10 000 permutations).

Blocks: with(df, Genotype)

Permutation: free

Number of permutations: 10000

adonis2(formula = d ~ Treatment + SamplingTimepoint + Tank, data = df, permutations = perm, method = "bray")

**Df SumOfSqs R2 F Pr(>F)**

Treatment 2 0.5223 0.01993 1.0045 0.2448

SamplingTimepoint 3 0.8291 0.03164 1.0631 0.1496

Tank 6 3.0189 0.11519 1.9354 0.6141

Residual 84 21.8380 0.83325

Total 95 26.2083 1.00000


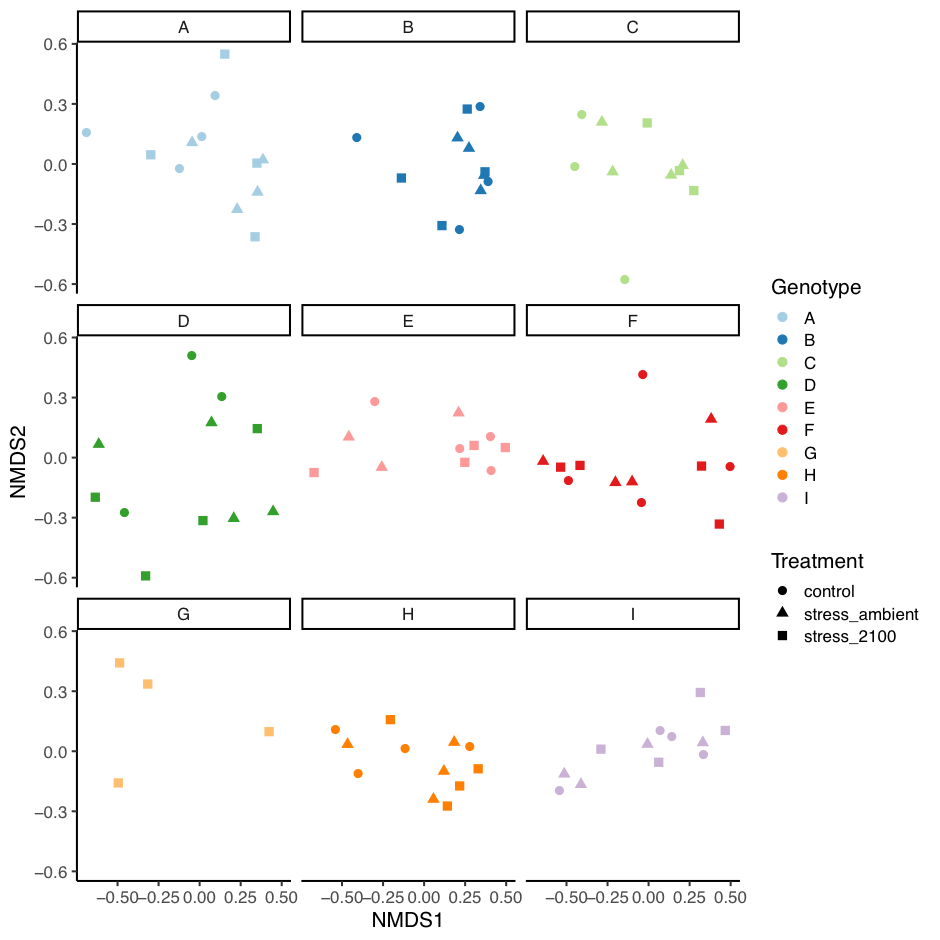


**Figure S1.** **Compositional stability of *Acropora tenuis* microbiome.** Non-metric multidimensional scaling (NMDS) plot based on unweighted unifrac distances (only considering the presence/absence of amplicon sequence variants).

**Figure S2.** **Microbiome composition of *Acropora tenuis*.** The relative abundance distribution of the 11 most abundant amplicon sequence variants (ASVs) associated with individual coral nubbins of each host genotype (A-I) under control, single stress and cumulative stress conditions over time (day 1, 10, 14 and 19).

**Table S4.** Statistical output of permutational ANOVA for db-RDA (anova.cca, vegan package) evaluating the contribution to the % explained observed community variation in the *A. tenuis* microbiome.

**Df SumOfSqs F Pr(>F)**

Genotype 8 6.9107 3.7584 0.000999 ***

Treatment 2 0.5902 1.2840 0.142857

SymbiontDensity 1 0.3569 1.5526 0.072927 .

Protein 1 0.1775 0.7725 0.716284

Chla 1 0.1945 0.8463 0.612388

Delta F/Fm’ 1 0.2474 1.0766 0.335664

Residual 81 18.6170

**Table S5.** Statistical output of PERMANOVA (adonis2, vegan package) testing the effect of treatment, sampling time point and tank on the *Endozoicomonas* community composition within each host genotype (10 000 permutations).

Blocks: with(df, Genotype)

Permutation: free

Number of permutations: 10000

adonis2(formula = d ~ Treatment + SamplingTimepoint + Tank, data = df, permutations = perm, method = "bray")

**Df SumOfSqs R2 F Pr(>F)**

Treatment 2 0.2114 0.01253 0.6081 0.7279

SamplingTimepoint 3 0.3192 0.01891 0.6120 0.7725

Tank 6 1.7420 0.10322 1.6699 0.7206

Residual 84 14.6045 0.86534

Total 95 16.8772 1.00000

**Table S6.** Indicator Value analysis output. Component ‘A’ is the probability that the surveyed site belongs to the target site group given the fact that the species has been found. This conditional probability is called the specificity or positive predictive value of the species as indicator of the site group. Component ‘B’ is the probability of finding the species in sites belonging to the site group. This second conditional probability is called the fidelity or sensitivity of the species as indicator of the target site group.

| **control** | **A** | **B** | **stat** | **p.value** | |
| --- | --- | --- | --- | --- | --- |
| Bacteria;Proteobacteria;Alphaproteobacteria;Rhodobacterales;Rhodobacteraceae;uncultured | 0.9678 | 0.1818 | 0.419 | 0.0214 | * |
| Bacteria;Bacteroidetes;Sphingobacteriia;Sphingobacteriales;Chitinophagaceae;Hydrotalea;uncultured bacterium | 1 | 0.1364 | 0.369 | 0.0272 | * |
| Bacteria;Proteobacteria;Alphaproteobacteria;Rhodobacterales;Rhodobacteraceae;Lentibacter;uncultured bacterium | 1 | 0.1364 | 0.369 | 0.0295 | * |
|  |  |  |  |  |  |
| **cumulative stress** | **A** | **B** | **stat** | **p.value** | |
| Bacteria;Proteobacteria;Alphaproteobacteria;Rhodobacterales;Rhodobacteraceae | 1 | 0.3077 | 0.555 | 0.0002 | *** |
| Bacteria;Proteobacteria;Alphaproteobacteria;Rhodobacterales;Rhodobacteraceae;Roseovarius | 1 | 0.2692 | 0.519 | 0.0011 | ** |
| Bacteria;Proteobacteria;Alphaproteobacteria;Rhodobacterales;Rhodobacteraceae | 1 | 0.2692 | 0.519 | 0.0011 | ** |
| Bacteria;Firmicutes;Clostridia;Clostridiales;Family XII;Fusibacter;uncultured bacterium | 1 | 0.1538 | 0.392 | 0.0289 | * |
| Bacteria;Bacteroidetes;Flavobacteriia;Flavobacteriales;Flavobacteriaceae | 1 | 0.1538 | 0.392 | 0.034 | * |
| Bacteria;Proteobacteria;Alphaproteobacteria;Rhodobacterales;Rhodobacteraceae;uncultured | 1 | 0.1538 | 0.392 | 0.034 | * |
|  |  |  |  |  |  |
| **single stress** | **A** | **B** | **stat** | **p.value** | |
| Bacteria | 1 | 0.2174 | 0.466 | 0.0052 | ** |
| Bacteria;Bacteroidetes;Flavobacteriia;Flavobacteriales;Cryomorphaceae;Owenweeksia;uncultured bacterium | 0.8976 | 0.2174 | 0.442 | 0.0185 | * |
| Bacteria;Proteobacteria;Alphaproteobacteria;Rhodobacterales;Rhodobacteraceae | 0.8835 | 0.2174 | 0.438 | 0.0291 | * |
| Bacteria | 1 | 0.1739 | 0.417 | 0.0172 | * |
| Bacteria;Proteobacteria;Gammaproteobacteria;Vibrionales;Vibrionaceae;Photobacterium;uncultured bacterium | 0.9604 | 0.1739 | 0.409 | 0.0329 | * |
| Bacteria;Proteobacteria;Alphaproteobacteria;Rhodobacterales;Rhodobacteraceae | 0.9472 | 0.1739 | 0.406 | 0.0298 | * |
| Bacteria;Proteobacteria;Alphaproteobacteria;Rhizobiales;Phyllobacteriaceae | 0.9348 | 0.1739 | 0.403 | 0.0427 | * |
| Bacteria;Proteobacteria;Alphaproteobacteria | 0.8777 | 0.1739 | 0.391 | 0.0458 | * |
| Bacteria;Proteobacteria;Alphaproteobacteria;Rhodobacterales;Rhodobacteraceae;Nautella;uncultured bacterium | 0.8726 | 0.1739 | 0.39 | 0.0447 | * |
| Bacteria;Planctomycetes;Planctomycetacia;Planctomycetales;Planctomycetaceae | 0.8701 | 0.1739 | 0.389 | 0.0354 | * |
| Bacteria | 0.8271 | 0.1739 | 0.379 | 0.0436 | * |
|  |  |  |  |  |  |
| **control + single stress** | **A** | **B** | **stat** | **p.value** | |
| Bacteria;Proteobacteria;Alphaproteobacteria;Rhodobacterales;Rhodobacteraceae;Ruegeria;uncultured bacterium | 0.9246 | 0.4444 | 0.641 | 0.0223 | * |
| Bacteria;Proteobacteria;Alphaproteobacteria;Rhodobacterales;Rhodobacteraceae;Ruegeria;uncultured bacterium | 0.8537 | 0.4222 | 0.6 | 0.0146 | * |
| Bacteria;Proteobacteria;Alphaproteobacteria;Rhodobacterales;Rhodobacteraceae;Ruegeria;uncultured bacterium | 1 | 0.3111 | 0.558 | 0.0042 | ** |
| Bacteria;Proteobacteria;Gammaproteobacteria;Enterobacteriales;Enterobacteriaceae;Escherichia-Shigella | 1 | 0.2 | 0.447 | 0.0431 | * |
|  |  |  |  |  |  |
| **single stress + cumulative stress** | **A** | **B** | **stat** | **p.value** | |
| Bacteria | 0.9301 | 0.3878 | 0.601 | 0.0408 | * |
| Bacteria;Proteobacteria;Gammaproteobacteria;Oceanospirillales;Hahellaceae;Endozoicomonas | 1 | 0.2245 | 0.474 | 0.0426 | * |

**Alpha diversity of the *Endozoicomonas* community associated with *A. tenuis* microbiome:**

Alpha diversity based on Shannon index (ANOVA, F_(8/87)_ = 2.091, p = 0.0452) and richness (ANOVA, F_(8/87)_ = 8.715, p = 1.1 x 10^-8^) varied significantly between host genotypes, however, remained stable throughout the experiment within each treatment (within subject ANOVA for Shannon index, F_(3/90)_ = 2.218, p = 0.0915; within subject ANOVA for richness, F_(3/90)_ = 2.388, p = 0.0741).
